# Supplementary material for: Is Composition of Brain Clot Retrieved by Mechanical Thrombectomy Associated with Stroke Aetiology and Clinical Outcomes in Acute Ischemic Stroke?—A Systematic Review and Meta-Analysis
Source: Neurol Int. 2022 Sep 20;14(4):748–70. doi: 10.3390/neurolint14040063 (PMC9589969; doi:10.3390/neurolint14040063)
Supplement: Supplementary file 1 [file neurolint-14-00063-s001.zip › neurolint-1919450-supplementary.pdf]

Supplementary Information (SI)

Is composition of brain clot retrieved by mechanical thrombectomy associated with stroke aetiology and clinical outcomes in acute ischemic stroke? – a systematic review and meta-analysis

List of contents

1. Search Strategy (Keywords/MeSH Terms)
2. PRISMA-2020 Checklist
3. Supplementary Figures
    a. Supplementary SI Figure S1. Influence of a single study in meta-analysis estimation: Clot Composition and Bridging Thrombolysis
    b. Supplementary SI Figure S2. Influence of a single study in meta-analysis estimation: RBC and Aetiology
    c. Supplementary SI Figure S3. Influence of a single study in meta-analysis estimation: Fibrin and Aetiology
    d. Supplementary SI Figure S4. Influence of a single study in meta-analysis estimation: Platelet and Aetiology
    e. Supplementary SI Figure S5. Influence of a single study in meta-analysis estimation: WBC and Aetiology
    f. Supplementary SI Figure S6. Influence of a single study in meta-analysis estimation: RBC and TICI Score
    g. Supplementary SI Figure S7. Influence of a single study in meta-analysis estimation: Clot Composition and Bridging Thrombolysis
4. Supplementary Tables
    a. Supplementary SI Table S1. Modified Jadad Analysis for Methodological Quality
    b. Supplementary SI Table S2. Baseline characteristics of studies excluded from meta-analysis
    c. Supplementary SI Table S3. Egger's test for publication bias assessment of the included studies
    d. Supplementary SI Table S4. Summary Effects of meta-analysis
5. References

1. Search Strategy:

MEDLINE/PubMed:

(("stroke" OR "ischemic stroke" OR "acute ischemic stroke" OR "AIS")
AND
("thrombus" OR "thrombi" OR "clot" OR "clots"))
AND
("thrombectomy" OR "endovascular" OR "clot retrieval" OR "mechanical thrombectomy" OR "EVT")
OR ("histology" OR "composition" OR "morphology")
Filters applied: Full text, Clinical Study, Clinical Trial, Clinical Trial Protocol, Clinical Trial, Phase I, Clinical Trial, Phase II, Clinical Trial, Phase III, Clinical Trial, Phase IV, Comparative Study, Controlled Clinical Trial, Dataset, Evaluation Study, Meta-Analysis, Multicenter Study, Observational Study, Pragmatic Clinical Trial, Randomized Controlled Trial, Review, Systematic Review, Validation Study, Humans, English, Adult: 19+ years

Google Scholar:

allintitle: ("stroke" OR "ischemic stroke" OR "acute ischemic stroke" OR "AIS" AND ("thrombus" OR "thrombi" OR "clot" OR "clots")
thrombectomy OR endovascular OR "clot retrieval" OR "mechanical thrombectomy" OR EVT OR histology OR composition
without: (“animal study”)

2. PRISMA-2020 Checklist

Section and Topic	Item #	Checklist item	Location where item is reported
**TITLE**			
Title	1	Identify the report as a systematic review.	1
**ABSTRACT**			
Abstract	2	See the PRISMA 2020 for Abstracts checklist.	3
**INTRODUCTION**			
Rationale	3	Describe the rationale for the review in the context of existing knowledge.	4
Objectives	4	Provide an explicit statement of the objective(s) or question(s) the review addresses.	5
**METHODS**			
Eligibility criteria	5	Specify the inclusion and exclusion criteria for the review and how studies were grouped for the syntheses.	5-6
Information sources	6	Specify all databases, registers, websites, organisations, reference lists and other sources searched or consulted to identify studies. Specify the date when each source was last searched or consulted.	5
Search strategy	7	Present the full search strategies for all databases, registers and websites, including any filters and limits used.	5, supplementary information
Selection process	8	Specify the methods used to decide whether a study met the inclusion criteria of the review, including how many reviewers screened each record and each report retrieved, whether they worked independently, and if applicable, details of automation tools used in the process.	6
Data collection process	9	Specify the methods used to collect data from reports, including how many reviewers collected data from each report, whether they worked independently, any processes for obtaining or confirming data from study investigators, and if applicable, details of automation tools used in the process.	6
Data items	10a	List and define all outcomes for which data were sought. Specify whether all results that were compatible with each outcome domain in each study were sought (e.g. for all	6

		measures, time points, analyses), and if not, the methods used to decide which results to collect.	
	10b	List and define all other variables for which data were sought (e.g. participant and intervention characteristics, funding sources). Describe any assumptions made about any missing or unclear information.	6
Study risk of bias assessment	11	Specify the methods used to assess risk of bias in the included studies, including details of the tool(s) used, how many reviewers assessed each study and whether they worked independently, and if applicable, details of automation tools used in the process.	6-7
Effect measures	12	Specify for each outcome the effect measure(s) (e.g. risk ratio, mean difference) used in the synthesis or presentation of results.	7
Synthesis methods	13a	Describe the processes used to decide which studies were eligible for each synthesis (e.g. tabulating the study intervention characteristics and comparing against the planned groups for each synthesis (item #5)).	6
	13b	Describe any methods required to prepare the data for presentation or synthesis, such as handling of missing summary statistics, or data conversions.	7
	13c	Describe any methods used to tabulate or visually display results of individual studies and syntheses.	6
	13d	Describe any methods used to synthesize results and provide a rationale for the choice(s). If meta-analysis was performed, describe the model(s), method(s) to identify the presence and extent of statistical heterogeneity, and software package(s) used.	7
	13e	Describe any methods used to explore possible causes of heterogeneity among study results (e.g. subgroup analysis, meta-regression).	7
	13f	Describe any sensitivity analyses conducted to assess robustness of the synthesized results.	7

Reporting bias assessment	14	Describe any methods used to assess risk of bias due to missing results in a synthesis (arising from reporting biases).	NA
Certainty assessment	15	Describe any methods used to assess certainty (or confidence) in the body of evidence for an outcome.	7
**RESULTS**			
Study selection	16a	Describe the results of the search and selection process, from the number of records identified in the search to the number of studies included in the review, ideally using a flow diagram.	7, 40
	16b	Cite studies that might appear to meet the inclusion criteria, but which were excluded, and explain why they were excluded.	Supplementary Information
Study characteristics	17	Cite each included study and present its characteristics.	8, 31
Risk of bias in studies	18	Present assessments of risk of bias for each included study.	Supplementary Information
Results of individual studies	19	For all outcomes, present, for each study: (a) summary statistics for each group (where appropriate) and (b) an effect estimate and its precision (e.g. confidence/credible interval), ideally using structured tables or plots.	8-14, Supplementary information
Results of syntheses	20a	For each synthesis, briefly summarise the characteristics and risk of bias among contributing studies.	8-14, supplementary information
	20b	Present results of all statistical syntheses conducted. If meta-analysis was done, present for each the summary estimate and its precision (e.g. confidence/credible interval) and measures of statistical heterogeneity. If comparing groups, describe the direction of the effect.	8-14
	20c	Present results of all investigations of possible causes of heterogeneity among study results.	NA
	20d	Present results of all sensitivity analyses conducted to assess the robustness of the synthesized results.	Supplementary Information
Reporting biases	21	Present assessments of risk of bias due to missing results (arising from reporting biases) for each synthesis assessed.	NA

Certainty of evidence	22	Present assessments of certainty (or confidence) in the body of evidence for each outcome assessed.	8-14
**DISCUSSION**			
Discussion	23a	Provide a general interpretation of the results in the context of other evidence.	15-17
	23b	Discuss any limitations of the evidence included in the review.	18
	23c	Discuss any limitations of the review processes used.	18
	23d	Discuss implications of the results for practice, policy, and future research.	19
**OTHER INFORMATION**			
Registration and protocol	24a	Provide registration information for the review, including register name and registration number, or state that the review was not registered.	NA
	24b	Indicate where the review protocol can be accessed, or state that a protocol was not prepared.	NA
	24c	Describe and explain any amendments to information provided at registration or in the protocol.	NA
Support	25	Describe sources of financial or non-financial support for the review, and the role of the funders or sponsors in the review.	20
Competing interests	26	Declare any competing interests of review authors.	20
Availability of data, code and other materials	27	Report which of the following are publicly available and where they can be found: template data collection forms; data extracted from included studies; data used for all analyses; analytic code; any other materials used in the review.	20

From: [1] Page MJ, McKenzie JE, Bossuyt PM, Boutron I, Hoffmann TC, Mulrow CD, et al. The PRISMA 2020 statement: an updated guideline for reporting systematic reviews. BMJ 2021;372:n71. doi: 10.1136/bmj.n71

3. Supplementary Figures

3a. Supplementary SI Figure S1. Influence of a single study in meta-analysis estimation: Clot Composition and Bridging Thrombolysis

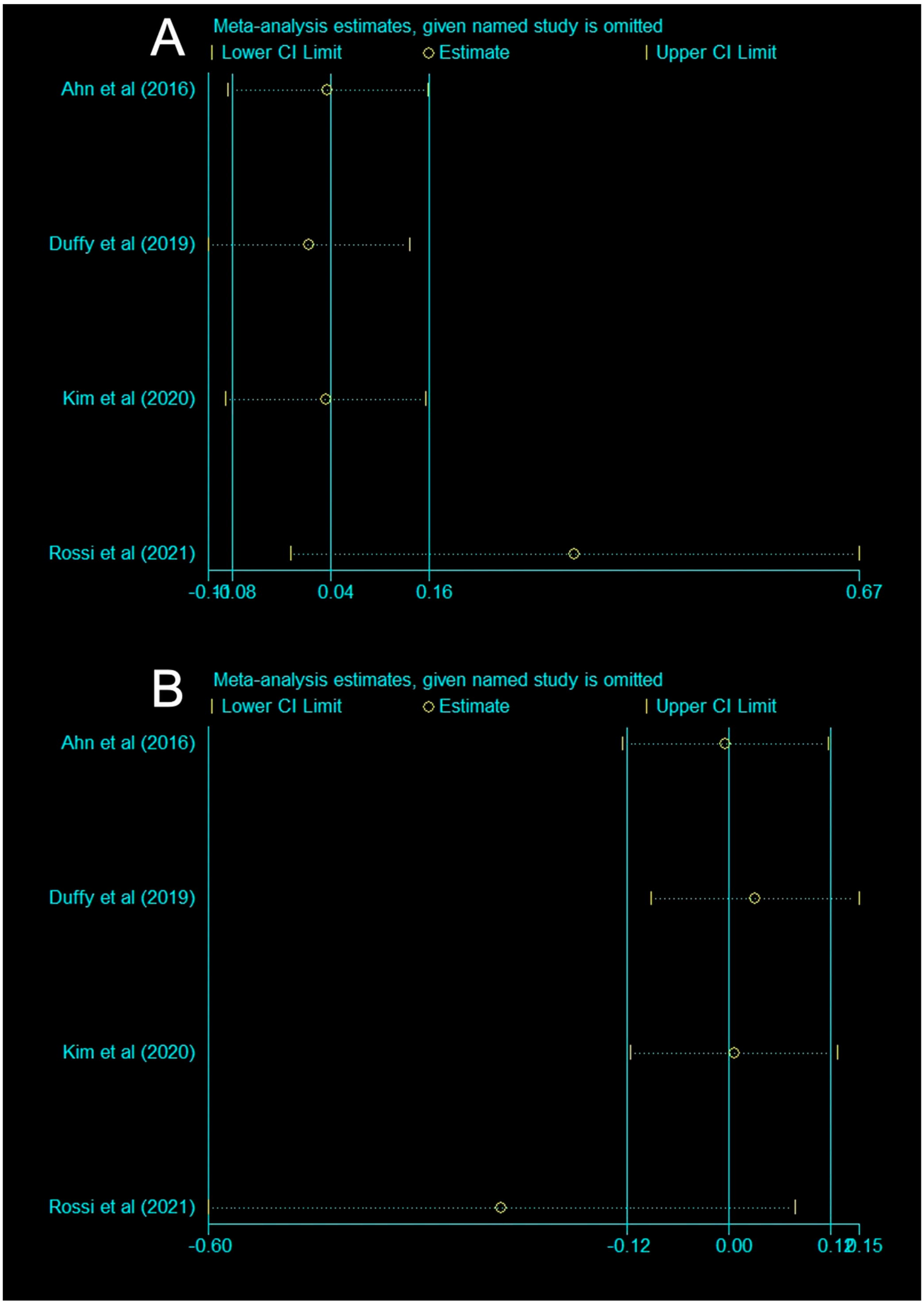

A: Red Blood Cell **(**RBC) Content and Bridging thrombolysis
B: Fibrin Content and Bridging Thrombolysis

3b. Supplementary SI Figure S2. Influence of a single study in meta-analysis estimation: RBC and Aetiology

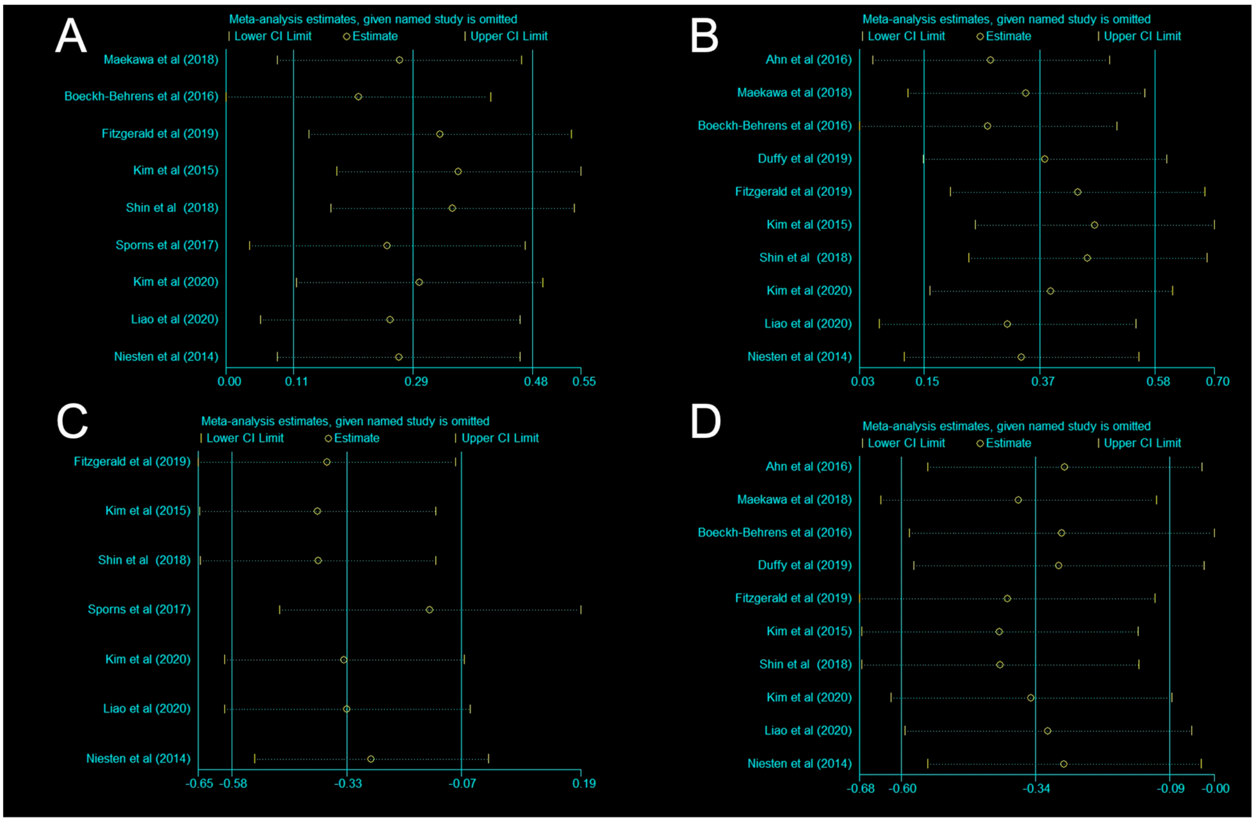

A: Non-cardioembolic vs Cardioembolic Stroke

B: Large artery atherosclerosis (LAA) vs Cardioembolic Stroke

C: Cryptogenic vs Non-cardioembolic stroke

D: Cryptogenic vs LAA stroke

3c. Supplementary SI Figure S3. Influence of a single study in meta-analysis estimation: Fibrin and Aetiology

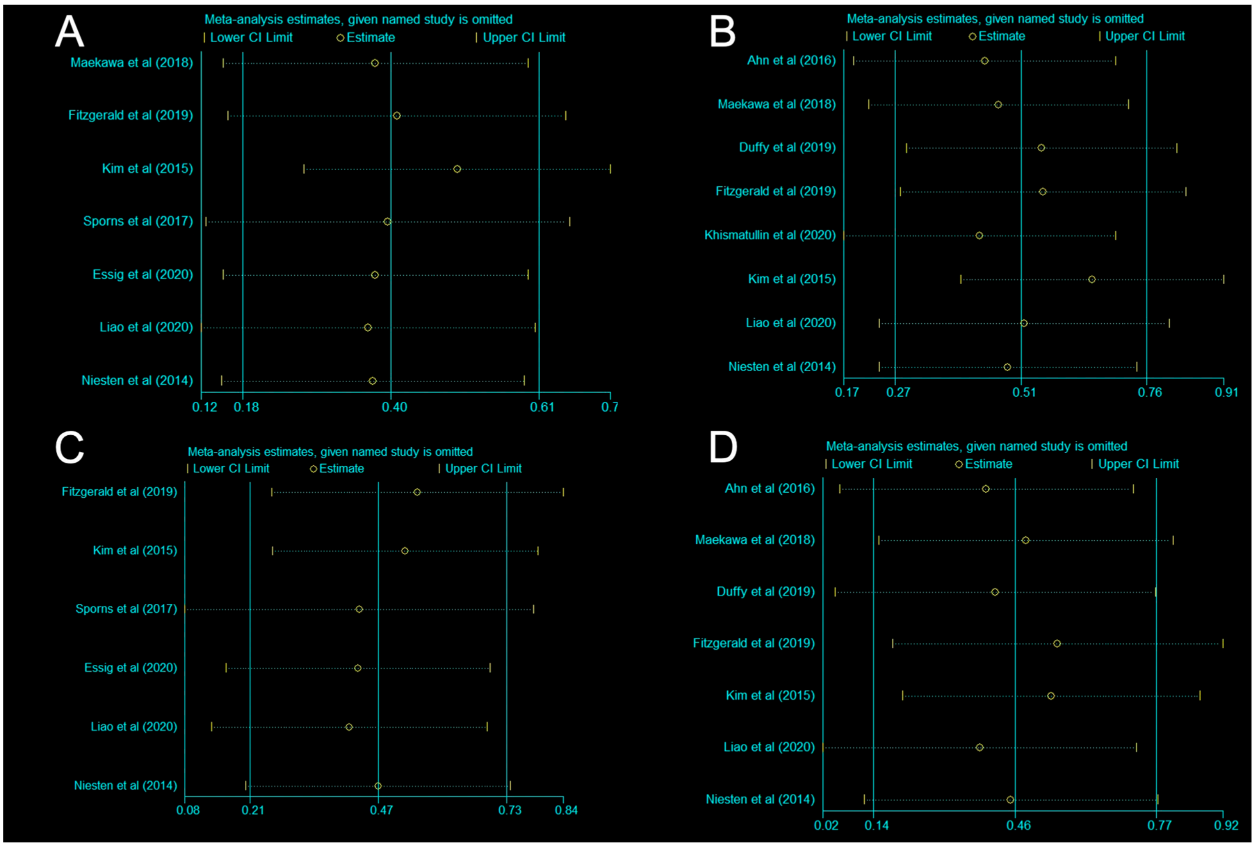

A: Cardioembolic vs non-cardioembolic stroke

B: Cardioembolic vs Large artery atherosclerosis (LAA) stroke

C: Cryptogenic vs non-cardioembolic stroke

D: Cryptogenic vs LAA Stroke

3d. Supplementary SI Figure S4. Influence of a single study in meta-analysis estimation: Platelet and Aetiology

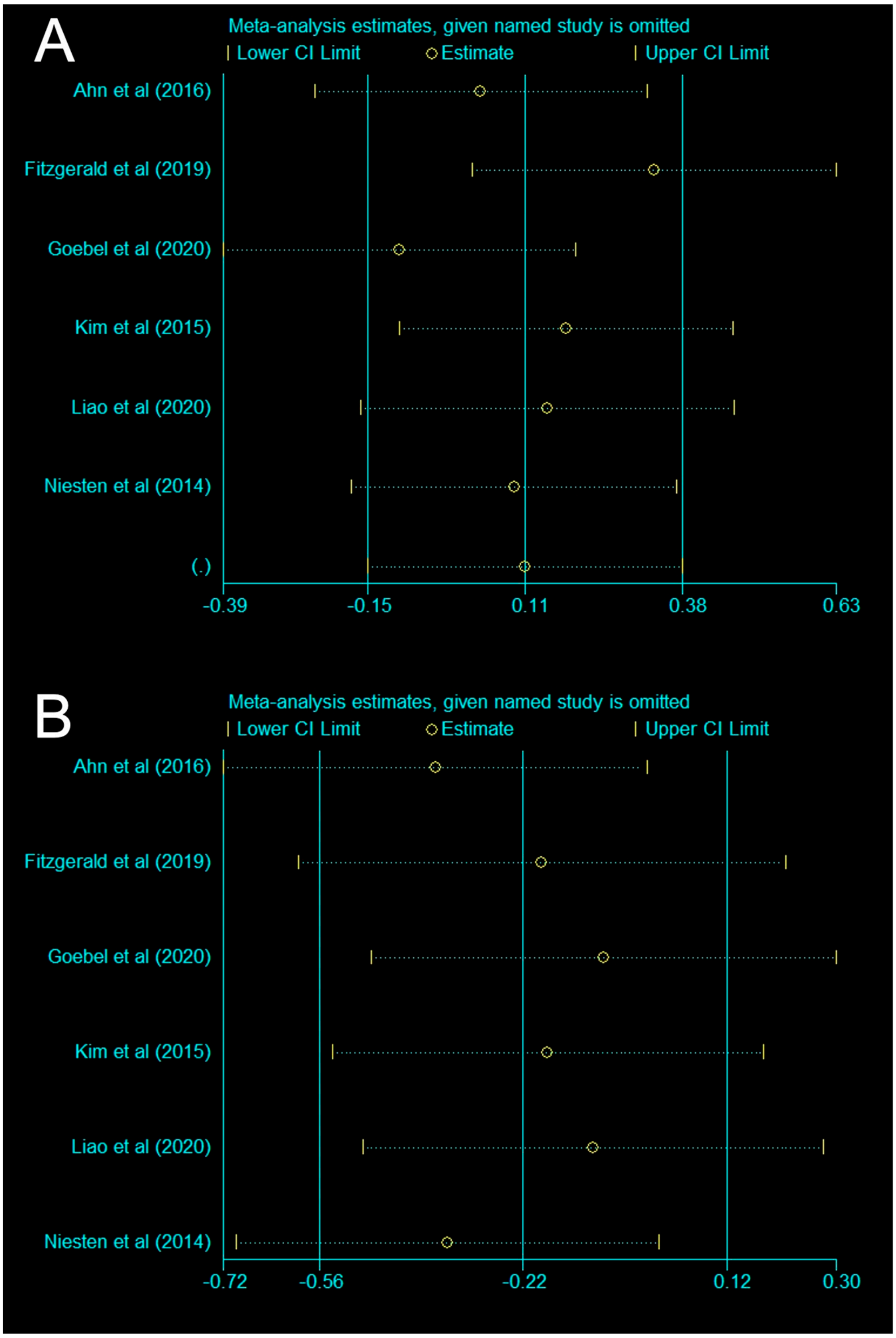

A: Cardioembolic vs Large artery atherosclerosis (LAA) Stroke

B: Cryptogenic vs LAA Stroke

3e. Supplementary SI Figure S5. Influence of a single study in meta-analysis estimation: WBC and Aetiology

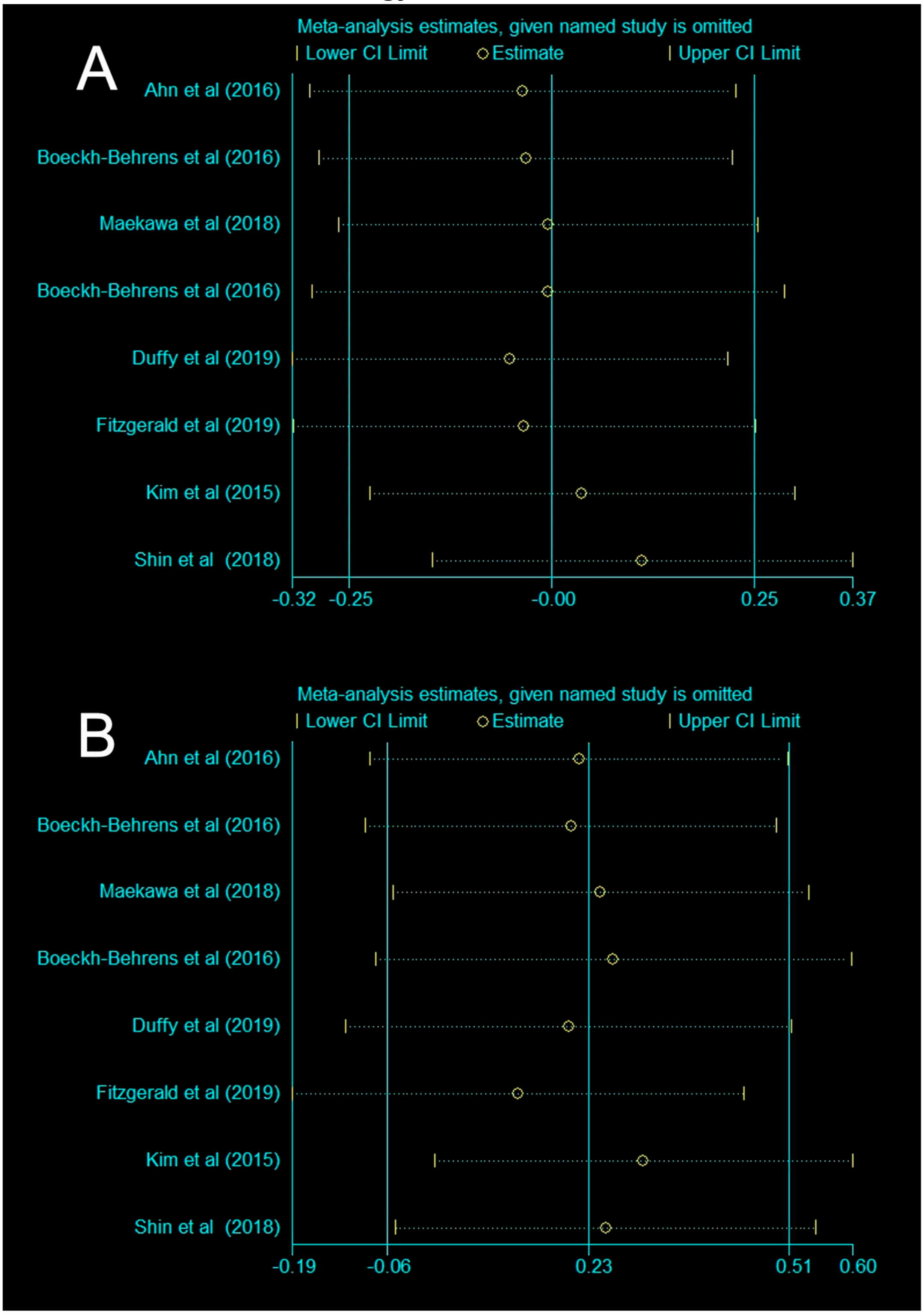

A: Cardioembolic vs Large artery atherosclerosis (LAA) Stroke

B: Cryptogenic vs LAA Stroke

3f. Supplementary SI Figure S6. Influence of a single study in meta-analysis estimation: RBC and TICI Score

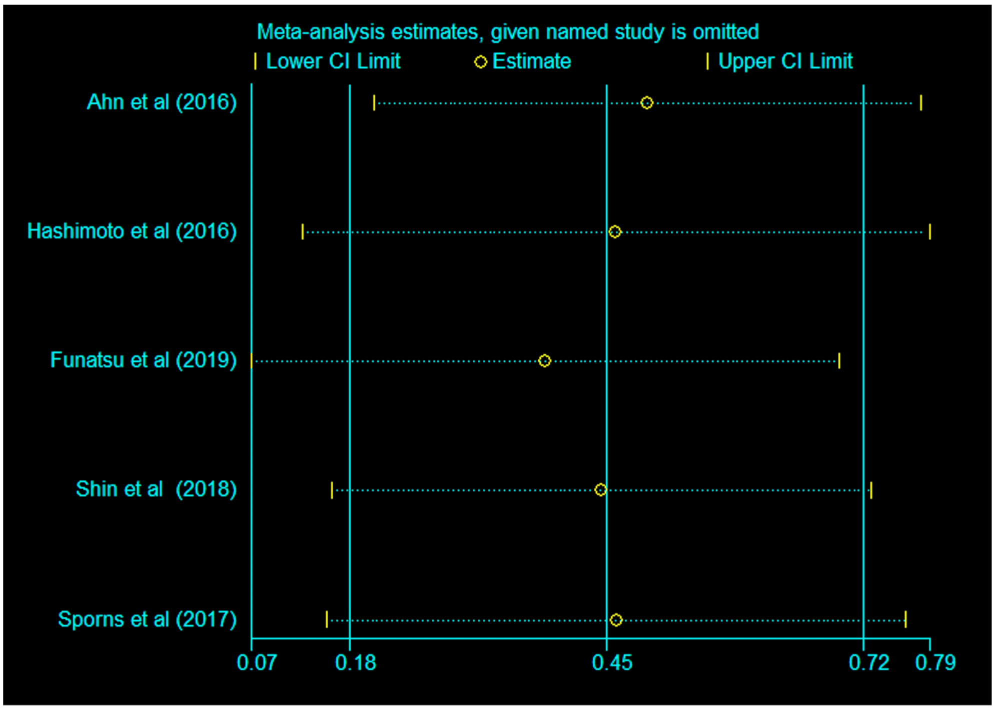

Abbreviations: CI: Confidence Interval, RBC: Red Blood Cell, TICI: Thrombolysis in Cerebral Infarction Scale

3g. Supplementary SI Figure S7. Influence of a single study in meta-analysis estimation: RBC and HMCAS

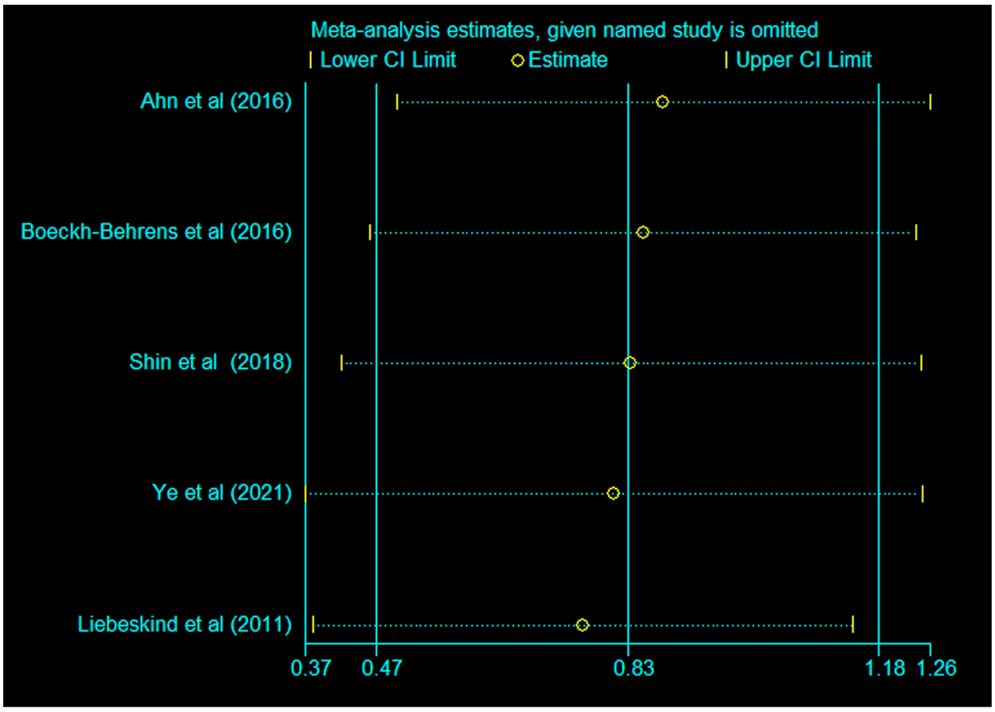

Abbreviations: CI: Confidence Interval, RBC: Red Blood Cell, HMCAS: Hyperdense Middle Cerebral Artery Sign

4. Supplementary Tables

4a. SI Table S1. Modified Jadad Analysis for Methodological Quality

Study ID	Study	Criteria 1	Criteria 2	Criteria 3	Criteria 4	Criteria 5	Criteria 6	Criteria 7	Criteria 8	Total	Publication Bias
**1**	Ahn et al (2016)[2]			✓	✓	✓	✓		✓	**5**	**0**
**2**	Boeckh-Behrens et al (2016a) [3]			✓	✓	✓	✓		✓	**5**	**0**
**3**	Hashimoto et al (2016) [4]			✓		✓	✓		✓	**5**	**0**
**4**	Maekawa et al (2018) [5]			✓	✓	✓	✓		✓	**5**	**0**
**5**	Boeckh-Behrens et al (2016a) [6]			✓	✓		✓		✓	**5**	**0**
**6**	Duffy et al (2019) [7]			✓			✓		✓	**3**	**1**
**7**	Fitzgerald et al						✓		✓	**2**	**1**

	(2019b) [8]										
**8**	Funatsu et al (2019) [9]			✓	✓		✓		✓	**4**	**0**
**9**	Goebel et al (2020) [10]					✓			✓	**2**	**1**
**10**	Khismatu llin et al (2020) [11]								✓	**1**	**0**
**11**	Kim et al (2015) [12]			✓	✓	✓	✓		✓	**5**	**0**
**12**	Shin et al (2018) [13]					✓	✓		✓	**3**	**0**
**13**	Sporns et al (2017a) [14]			✓	✓		✓	✓	✓	**5**	**0**
**14**	Sporns et al (2017b) [15]			✓	✓		✓		✓	**4**	**0**
**15**	Ye et al (2021) [16]						✓	✓	✓	**3**	**0**

**16**	Essig et al (2020) [17]						✓		✓	**2**	**1**
**17**	Kim et al (2020) [18]			✓	✓		✓		✓	**4**	**0**
**18**	Liao et al (2020) [19]			✓	✓	✓	✓		✓	**5**	**0**
**19**	Niesten et al (2014) [20]			✓	✓		✓		✓	**4**	**0**
**20**	Liebeskind et al (2011) [21]			✓	✓		✓		✓	**4**	**1**
**21**	Rossi et al (2021) [22]						✓		✓	**2**	**0**

Criteria 1 = Was the study randomised?
Criteria 2 = Was the method of randomisation appropriate? (not specified = 0)
Criteria 3 = Was the study described as being blinded?
Criteria 4 = Was the method of blinding appropriate?
Criteria 5 = Was there a description of withdrawals and dropouts?
Criteria 6 = Was there a clear description of the inclusion/exclusion criteria?
Criteria 7 = Was the method used to assess adverse events described?
Criteria 8 = Was the method of statistical analysis described?
Publication Bias (0 = no grants, 1: any conflicts of interest declared relating to industry funding outside of the current research publication; 2: if the study had industry funding)

4b. SI Table S2. Baseline Characteristics of Studies For Systematic Review and Reason For Exclusion From Meta-analysis

Study	Design	No. of Centres	Cohort size	Mean Age (SD)	Male, n (%)	Histological Staining Method(s)	Thrombectomy Device(s)	TICI 2b-3, n (%)	HMCAS +, n (%)	IVT, n (%)	RBC% Mean (SD)	TOAST, n				Reason for Exclusion
												**1**	**2**	**4**	**5**	
Gong et al (2019) [23]	Retrospective Cohort	1	45			H&E	Solitaire AB stent retriever				69 (*)	9	36			Data was unextractable
Simons et al (2015) [24]	Prospective Cohort	1	40	65.6 (12.9)	27 (68))	H&E, CD34	Solitaire FR Revascularization Device		29/39[a] (28)	28 (70)		19 [b]	21			Clots categorised as RBC-rich or poor rather than continuous data
Douglas et al (2020) [25]	Retrospective Cohort	1	63			MSB, CD42b, vWF		59 (94)		28 (44)	39.2 (*)	9	20	2	32	Data was unextractable
Prochazka et al (2018) [26]	Prospective Cohort	1	131		69 (53)	H&E, vWF, CD31, CD15, Carstairs	Penumbra aspiration device, Solitaire SR, Catch SR, pRESET SR			101 (77)						Data was unextractable

Singh et al (2013) [27]	Retrospective Cohort	1	48	67 (43-91) [c]	29 (60)	H&E, Prussian Blue, EVG, Kossa, Periodic acid-Schiff reaction, CD34	Solitaire FR, Aperio device	35 (73)								Data was unextractable
Fitzgerald et al (2019b) [28]	Retrospective Cohort	2	50	67 (20-91)[c]	28 (56)	H&E		49 (98)		23 (46)		11	30	5	4	Data was unextractable
Fitzgerald et al (2019c) [29]	Retrospective Cohort	>1	85			H&E, MSB			82 (96)	43 (51)		19	44	9	13	SDs were not reported
Horie et al (2019) [30]	Retrospective Cohort	1	75		31 (41)	H&E	Penumbra suction catheter ADAPT, Solitaire SR, Trevo SR, BGC	51 (68)		22 (29)		21[b]	54			Data was unextractable
Kaesmacher et al (2017) [31]	Retrospective Cohort	1	85	70.2 (14.6)	40 (47)	H&E, neutrophil elastase	Solitaire, Trevo, Revive, Pulse, pREset 4 –20		57 (67)	58 (68)	37 (*)					Data was unextractable

Abbreviations: TICI: Thrombolysis In Cerebral Infarction, HMCAS: Hyperdense Middle Cerebral Artery Sign, IVT: Intravenous Thrombolysis, RBC: Red Blood Cell, TOAST: Trial of Org 10172 in Acute Stroke Treatment, H&E: Haematoxylin and Eosin, MSB: Martius Scarlet Blue, vWF: von Willebrand Factor, EVG: Elastica van Gieson

*Unavailable

[a] Data was not available for all patients

[b] Number of non-cardioembolic thrombi reported

[c] Age reported as median (range) where mean (SD) was not available

4c. Supplementary SI Table S3. Egger's test for publication bias assessment of the included studies

Meta-Analysis	Slope		Bias		Test of H0: no small-study effects	Publication Bias
	Coefficient (95% CI)	Standard Error	Coefficient (95%CI)	Standard Error	P-value	Yes/No
RBC Aetiology (non-CE vs CE)	0.92 (-0.64 – 2.49)	0.66	1.46 (-0.20 – 3.11)	0.70	0.076	Yes
RBC Aetiology (LAA vs CE)	1.76 (0.03 – 3.49)	0.75	2.46 (0.35 – 4.56)	0.91	0.028	No
Fibrin Aetiology (CE vs non-CE)	0.57 (-0.12 – 1.02)	0.17	0.92 (0.43 – 1.42)	0.19	0.005	No

Fibrin Aetiology (CE vs LAA)	0.92 (-0.27 – 2.11)	0.49	1.27 (-0.15 – 1.22)	0.58	0.071	Yes
Platelet Aetiology	0.01 (-3.93 – 3.94)	1.42	0.99 (-3.32 – 5.30)	1.55	0.558	Yes
WBC Aetiology	1.03 (-1.83 – 3.89)	1.17	2.83 (-1.83 – 3.89)	1.51	0.110	Yes
RBC Cryptogenic vs non-CE	1.00 (-0.44 – 2.44)	0.56	1.93 (-0.15 – 3.87)	0.75	0.051	Yes
RBC Cryptogenic vs LAA	1.17 (-0.12 – 2.46)	0.56	2.33 (0.25 – 4.40)	0.90	0.032	No
Fibrin Cryptogenic vs non-CE	-0.21 (-2.51 – 2.09)	0.83	0.53 (-2.41 – 3.47)	1.06	0.643	Yes
Fibrin Cryptogenic vs LAA	-0.55 (-3.51 – 2.41)	1.15	0.35 (-4.10 – 4.80)	1.73	0.846	Yes
Platelet Cryptogenic	1.14 (0.11 – 2.19)	0.37	1.88 (0.24 – 3.51)	0.59	0.034	No

WBC Cryptogenic	-0.59 (-3.69 – 2.51)	1.27	1.09 (-3.87 – 2.51)	2.03	0.611	Yes
RBC TICI	-1.79 (-6.81 – 3.23)	1.58	-0.71 (-5.52 – 4.09)	1.51	0.669	Yes
RBC HMCAS	-0.98 (-4.10 – 04.75)	0.98	-0.84 (-4.75 – 3.07)	1.23	0.543	Yes
RBC IVT	1.82 (-6.35 – 9.98)	1.90	2.77 (-3.34 – 8.88)	1.95	0.191	Yes
Fibrin IVT	-0.74 (-8.60 – 7.12)	1.83	0.73 (-5.15 – 6.62)	1.37	0.645	Yes

Abbreviations: RBC = red blood cell, CE = cardioembolic, LAA = large artery atherosclerosis, TICI = thrombolysis in cerebral infarction, HMCAS = hyperdense middle cerebral artery sign, IVT = intravenous thrombolysis.

4d. SI Table S4. Summary Effects of meta-analysis

Outcome	Groups	Summary Effects	Test of Overall Effect	Heterogeneity and Variance Estimates			
				Cochran's Q	I^2 (%)	p-value	tau^2
**RBC Content**	Non-CE vs CE	SMD = 0.184; 95% CI, -0.191 – 0.558	z = 1.425, p = 0.154	28.10	71.5	<0.001	0.2135
	LAA vs CE	SMD = 0.368; 95% CI, -0.138 – 0.874	z = 0.961, p = 0.337	45.02	80.0	<0.001	0.5067
	Cryptogenic vs non-CE	SMD = -0.232; 95% CI, -0.651 – 0.188	z = -1.081, p = 0.280	13.39	55.2	0.037	0.1615
	Cryptogenic vs LAA	SMD = -0.336; 95% CI, -0.738 – 0.065	z = -1.644, p = 0.100	19.85	54.7	0.019	0.2133
	TICI2b-3 vs TICI0-2a	SMD= 0.450; 95% CI, 0.177 – 0.722	z = 3.237, p = 0.001	1.20	<0.1	0 878	<0.0001
	HMCAS+ vs HMCAS-	SMD = 0.827; 95% CI, 0.472 – 1.183	z = 4.564, p <0.001	1.28	<0.1	0.865	<0.0001
	IVT+ vs IVT-	SMD = 0.138; 95% CI, -0.109 – 0.385	z = 1.093, p = 0.274	4.45	32.6	0.217	0.0234
**Fibrin Content**	CE vs non-CE	SMD = 0.388; 95% CI, 0.032 – 0.745	z = 2.135, p = 0.033	13.63	56.0	0.034	0.1169
	CE vs LAA	SMD = 0.552; 95% CI, 0.099 – 1.004	z = 2.388, p = 0.017	21.84	67.9	0.003	0.2749

	Cryptogenic vs non-CE	SMD = 0.468; 95% CI, 0.172 – 0.765	z = 3.097, p = 0.002	5.78	13.4	0.329	0.0194
	Cryptogenic vs LAA	SMD = 0.455; 95% CI, 0.137 – 0.774	z = 2.805, p = 0.005	6.03	0.5	0.420	0.0009
	IVT+ vs IVT-	SMD = -0.109; 95% CI, -0.403 – 0.186	z = -0.723, p = 0.470	5.60	46.5	0.133	0.0421
**Platelet Content**	CE vs LAA	SMD = 0.168; 95% CI, -0.360 – 0.696	z = 0.623, p = 533	18.44	72.9	0.002	0.3034
	Cryptogenic vs LAA	SMD = -0.001; 95% CI, -0.669 – 0.666	z = -0.004, p = -.997	17.67	71.7	0.003	0.4765
**WBC Content**	CE vs LAA	SMD= -0.028; 95% CI, -0.394 – 0.338	z = -0.148, p = 0.882	13.51	48.2	0.061	0.1266
	Cryptogenic vs LAA	SMD = 0.227; 95% CI, -0.057 – 0.511	z = 1.568, p = 0.117	6.33	<0.1	0.502	<0.0001

Abbreviations: RBC: Red Blood Cell, CE: Cardioembolic, Non-CE: Non-cardioembolic, LAA: Large Artery Atherosclerosis, TICI: Thrombolysis in Cerebral Infarction, HMCAS: Hyperdense Middle Cerebral Artery Sign, IVT: Intravenous Thrombolysis, WBC: White Blood Cell, SMD = Standard Mean Difference, CI = Confidence Interval

6. References

1. Page, M.J.; McKenzie, J.E.; Bossuyt, P.M.; Boutron, I.; Hoffmann, T.C.; Mulrow, C.D.; Shamseer, L.; Tetzlaff, J.M.; Akl, E.A.; Brennan, S.E.; et al. The PRISMA 2020 statement: an updated guideline for reporting systematic reviews. *BMJ* **2021**, *372*, n71, doi:10.1136/bmj.n71.
2. Ahn, S.H.; Hong, R.; Choo, I.S.; Heo, J.H.; Nam, H.S.; Kang, H.G.; Kim, H.W.; Kim, J.H. Histologic features of acute thrombi retrieved from stroke patients during mechanical reperfusion therapy. *Int J Stroke* **2016**, *11*, 1036-1044, doi:10.1177/1747493016641965.
3. Boeckh-Behrens, T.; Schubert, M.; Förschler, A.; Prothmann, S.; Kreiser, K.; Zimmer, C.; Riegger, J.; Bauer, J.; Neff, F.; Kehl, V.; et al. The Impact of Histological Clot Composition in Embolic Stroke. *Clin Neuroradiol* **2016**, *26*, 189-197, doi:10.1007/s00062-014-0347-x.
4. Hashimoto, T.; Hayakawa, M.; Funatsu, N.; Yamagami, H.; Satow, T.; Takahashi, J.C.; Nagatsuka, K.; Ishibashi-Ueda, H.; Kira, J.I.; Toyoda, K. Histopathologic Analysis of Retrieved Thrombi Associated With Successful Reperfusion After Acute Stroke Thrombectomy. *Stroke* **2016**, *47*, 3035-3037, doi:10.1161/strokeaha.116.015228.
5. Maekawa, K.; Shibata, M.; Nakajima, H.; Mizutani, A.; Kitano, Y.; Seguchi, M.; Yamasaki, M.; Kobayashi, K.; Sano, T.; Mori, G.; et al. Erythrocyte-Rich Thrombus Is Associated with Reduced Number of Maneuvers and Procedure Time in Patients with Acute Ischemic Stroke Undergoing Mechanical Thrombectomy. *Cerebrovasc Dis Extra* **2018**, *8*, 39-49, doi:10.1159/000486042.
6. Boeckh-Behrens, T.; Kleine, J.F.; Zimmer, C.; Neff, F.; Scheipl, F.; Pelisek, J.; Schirmer, L.; Nguyen, K.; Karatas, D.; Poppert, H. Thrombus Histology Suggests Cardioembolic Cause in Cryptogenic Stroke. *Stroke* **2016**, *47*, 1864-1871, doi:doi:10.1161/STROKEAHA.116.013105.
7. Duffy, S.; McCarthy, R.; Farrell, M.; Thomas, S.; Brennan, P.; Power, S.; O’Hare, A.; Morris, L.; Rainsford, E.; MacCarthy, E.; et al. Per-Pass Analysis of Thrombus Composition in Patients With Acute Ischemic Stroke Undergoing Mechanical Thrombectomy. *Stroke* **2019**, *50*, 1156-1163, doi:doi:10.1161/STROKEAHA.118.023419.
8. Fitzgerald, S.; Dai, D.; Wang, S.; Douglas, A.; Kadirvel, R.; Layton, K.F.; Thacker, I.C.; Gounis, M.J.; Chueh, J.-Y.; Puri, A.S.; et al. Platelet-Rich Emboli in Cerebral Large Vessel Occlusion Are Associated With a Large Artery Atherosclerosis Source. *Stroke* **2019**, *50*, 1907-1910, doi:doi:10.1161/STROKEAHA.118.024543.
9. Funatsu, N.; Hayakawa, M.; Hashimoto, T.; Yamagami, H.; Satow, T.; Takahashi, J.C.; Koga, M.; Nagatsuka, K.; Ishibashi-Ueda, H.; Iwama, T.; et al. Vascular wall components in thrombi obtained by acute stroke thrombectomy: clinical significance and related factors. *Journal of NeuroInterventional Surgery* **2019**, *11*, 232, doi:10.1136/neurintsurg-2018-014041.
10. Goebel, J.; Gaida, B.J.; Wanke, I.; Kleinschnitz, C.; Koehrmann, M.; Forsting, M.; Moenninghoff, C.; Radbruch, A.; Junker, A. Is Histologic Thrombus Composition in Acute Stroke Linked to Stroke Etiology or to Interventional Parameters? *American Journal of Neuroradiology* **2020**, *41*, 650, doi:10.3174/ajnr.A6467.
11. Khismatullin, R.R.; Nagaswami, C.; Shakirova, A.Z.; Vrtková, A.; Procházka, V.; Gumulec, J.; Mačák, J.; Litvinov, R.I.; Weisel, J.W. Quantitative Morphology of Cerebral Thrombi Related to Intravital Contraction and Clinical Features of Ischemic Stroke. *Stroke* **2020**, *51*, 3640-3650, doi:doi:10.1161/STROKEAHA.120.031559.

12. Kim, S.; Yoon, W.; Kim, T.; Kim, H.; Heo, T.; Park, M. Histologic analysis of retrieved clots in acute ischemic stroke: correlation with stroke etiology and gradient-echo MRI. *American Journal of Neuroradiology* **2015**, *36*, 1756-1762.
13. Shin, J.W.; Jeong, H.S.; Kwon, H.-J.; Song, K.S.; Kim, J. High red blood cell composition in clots is associated with successful recanalization during intra-arterial thrombectomy. *PLoS One* **2018**, *13*, e0197492.
14. Sporns, P.B.; Hanning, U.; Schwindt, W.; Velasco, A.; Buerke, B.; Cnyrim, C.; Minnerup, J.; Heindel, W.; Jeibmann, A.; Niederstadt, T. Ischemic Stroke: Histological Thrombus Composition and Pre-Interventional CT Attenuation Are Associated with Intervention Time and Rate of Secondary Embolism. *Cerebrovascular Diseases* **2017**, *44*, 344-350, doi:10.1159/000481578.
15. Sporns, P.B.; Hanning, U.; Schwindt, W.; Velasco, A.; Minnerup, J.; Zoubi, T.; Heindel, W.; Jeibmann, A.; Niederstadt, T.U. Ischemic Stroke: What Does the Histological Composition Tell Us About the Origin of the Thrombus? . *Stroke* **2017**, *48*, 2206-2210, doi:doi:10.1161/STROKEAHA.117.016590.
16. Ye, G.; Cao, R.; Lu, J.; Qi, P.; Hu, S.; Chen, K.; Tan, T.; Chen, J.; Wang, D. Histological composition behind CT-based thrombus density and perviousness in acute ischemic stroke. *Clinical Neurology and Neurosurgery* **2021**, *207*, 106804, doi:https://doi.org/10.1016/j.clineuro.2021.106804.
17. Essig, F.; Kollikowski, A.M.; Pham, M.; Solymosi, L.; Stoll, G.; Haeusler, K.G.; Kraft, P.; Schuhmann, M.K. Immunohistological analysis of neutrophils and neutrophil extracellular traps in human thrombemboli causing acute ischemic stroke. *International journal of molecular sciences* **2020**, *21*, 7387.
18. Kim, B.; Kim, Y.M.; Jin, S.-C.; Lee, J.-w.; Lee, B.I.; Kim, S.E.; Shin, K.J.; Park, J.; Park, K.M.; Kim, S.E. Development of a predictive scale for cardioembolic stroke using extracted thrombi and angiographic findings. *Journal of Clinical Neuroscience* **2020**, *73*, 224-230.
19. Liao, Y.; Guan, M.; Liang, D.; Shi, Y.; Liu, J.; Zeng, X.; Huang, S.; Xie, X.; Yuan, D.; Qiao, H. Differences in pathological composition among large artery occlusion cerebral thrombi, Valvular heart disease atrial thrombi and carotid Endarterectomy plaques. *Frontiers in Neurology* **2020**, 811.
20. Niesten, J.M.; van der Schaaf, I.C.; van Dam, L.; Vink, A.; Vos, J.A.; Schonewille, W.J.; de Bruin, P.C.; Mali, W.P.T.M.; Velthuis, B.K. Histopathologic Composition of Cerebral Thrombi of Acute Stroke Patients Is Correlated with Stroke Subtype and Thrombus Attenuation. *PLOS ONE* **2014**, *9*, e88882, doi:10.1371/journal.pone.0088882.
21. Liebeskind, D.S.; Sanossian, N.; Yong, W.H.; Starkman, S.; Tsang, M.P.; Moya, A.L.; Zheng, D.D.; Abolian, A.M.; Kim, D.; Ali, L.K.; et al. CT and MRI Early Vessel Signs Reflect Clot Composition in Acute Stroke. *Stroke* **2011**, *42*, 1237-1243, doi:doi:10.1161/STROKEAHA.110.605576.
22. Rossi, R.; Molina, S.; Mereuta, O.M.; Douglas, A.; Fitzgerald, S.; Tierney, C.; Pandit, A.; Brennan, P.; Power, S.; O'Hare, A.; et al. Does prior administration of rtPA influence acute ischemic stroke clot composition? Findings from the analysis of clots retrieved with mechanical thrombectomy from the RESTORE registry. *J Neurol* **2022**, *269*, 1913-1920, doi:10.1007/s00415-021-10758-5.
23. Gong, L.; Zheng, X.; Feng, L.; Zhang, X.; Dong, Q.; Zhou, X.; Wang, H.; Zhang, X.; Shu, Z.; Zhao, Y.; et al. Bridging Therapy Versus Direct Mechanical Thrombectomy in Patients with Acute Ischemic Stroke due to Middle Cerebral Artery Occlusion: A Clinical-Histological Analysis of Retrieved Thrombi. *Cell Transplant* **2019**, *28*, 684-690, doi:10.1177/0963689718823206.

24. Simons, N.; Mitchell, P.; Dowling, R.; Gonzales, M.; Yan, B. Thrombus composition in acute ischemic stroke: A histopathological study of thrombus extracted by endovascular retrieval. *Journal of Neuroradiology* **2015**, *42*, 86-92, doi:https://doi.org/10.1016/j.neurad.2014.01.124.
25. Douglas, A.; Fitzgerald, S.; Mereuta, O.M.; Rossi, R.; Leary, S.; Pandit, A.; McCarthy, R.; Gilvarry, M.; Holmegaard, L.; Abrahamsson, M.; et al. Platelet-rich emboli are associated with von Willebrand factor levels and have poorer revascularization outcomes. *Journal of NeuroInterventional Surgery* **2020**, *12*, 557, doi:10.1136/neurintsurg-2019-015410.
26. Prochazka, V.; Jonszta, T.; Czerny, D.; Krajca, J.; Roubec, M.; Macak, J.; Kovar, P.; Kovarova, P.; Pulcer, M.; Zoubkova, R.; et al. The Role of von Willebrand Factor, ADAMTS13, and Cerebral Artery Thrombus Composition in Patient Outcome Following Mechanical Thrombectomy for Acute Ischemic Stroke. *Med. Sci. Monit.* **2018**, *24*, 3929-3945, doi:10.12659/MSM.908441.
27. Singh, P.; Doostkam, S.; Reinhard, M.; Ivanovas, V.; Taschner, C.A. Immunohistochemical Analysis of Thrombi Retrieved During Treatment of Acute Ischemic Stroke. *Stroke* **2013**, *44*, 1720-1722, doi:doi:10.1161/STROKEAHA.113.000964.
28. Fitzgerald, S.; Wang, S.; Dai, D.; Murphree Jr, D.H.; Pandit, A.; Douglas, A.; Rizvi, A.; Kadirvel, R.; Gilvarry, M.; McCarthy, R. Orbit image analysis machine learning software can be used for the histological quantification of acute ischemic stroke blood clots. *PloS one* **2019**, *14*, e0225841.
29. Fitzgerald, S.T.; Wang, S.; Dai, D.; Douglas, A.; Kadirvel, R.; Gounis, M.J.; Chueh, J.; Puri, A.S.; Layton, K.F.; Thacker, I.C.; et al. Platelet-rich clots as identified by Martius Scarlet Blue staining are isodense on NCCT. *Journal of NeuroInterventional Surgery* **2019**, *11*, 1145, doi:10.1136/neurintsurg-2018-014637.
30. Horie, N.; Shobayashi, K.; Morofuji, Y.; Sadakata, E.; Iki, Y.; Matsunaga, Y.; Kanamoto, T.; Tateishi, Y.; Izumo, T.; Anda, T.; et al. Impact of Mechanical Thrombectomy Device on Thrombus Histology in Acute Embolic Stroke. *World Neurosurgery* **2019**, *132*, e418-e422, doi:https://doi.org/10.1016/j.wneu.2019.08.130.
31. Kaesmacher, J.; Boeckh-Behrens, T.; Simon, S.; Maegerlein, C.; Kleine, J.; Zimmer, C.; Schirmer, L.; Poppert, H.; Huber, T. Risk of thrombus fragmentation during endovascular stroke treatment. *American Journal of Neuroradiology* **2017**, *38*, 991-998.
